# Sex difference in IgE sensitization associated with alcohol consumption in the general population

**DOI:** 10.1038/s41598-019-48305-y

**Published:** 2019-08-20

**Authors:** Daeyoung Roh, Dong-Hee Lee, Sang-Kyu Lee, Soo Whan Kim, Sung Won Kim, Jin Hee Cho, Byung-Guk Kim, Ji-Hyeon Shin

**Affiliations:** 1 Mind-neuromodulation Laboratory and Department of Psychiatry, Chuncheon Sacred Heart Hospital, Hallym University College of Medicine, Chuncheon-si, Gangwon-do Republic of Korea; 2 Department of Otolaryngology-Head and Neck Surgery, College of Medicine, The Catholic University of Korea, Seoul, Republic of Korea

**Keywords:** Disease prevention, Epidemiology, Risk factors

## Abstract

The association of alcohol consumption and immunoglobulin E (IgE) sensitization is debated. Few population-based studies have investigated whether such associations differ by sex. We explored the association of alcohol consumption with IgE sensitization in the general population, stratified by sex. We analyzed data for 1,723 adults from the 2010 Korean National Health and Nutrition Examination Survey. We divided subjects into three groups according to their self-reported alcohol consumption or serum level of gamma-glutamyltransferase (GGT), an objective marker of alcohol consumption. After adjustments, the odds ratios (ORs) of male high-risk drinkers were 2.09 (95% confidence interval [CI], 1.34–3.28) for total IgE and 1.71 (95% CI, 1.03–2.83) for *Dermatophagoides farinae* (DF)-specific IgE compared with male low-risk drinkers. In females, the dog-specific IgE level was associated with high-risk drinking (OR, 11.74; 95% CI, 2.04–67.24). The ORs of males in the high-serum-GGT group were 2.73 (95% CI, 1.72–4.33) for total IgE and 2.17 (95% CI, 1.35–3.47) for DF-specific IgE compared with those in the low-serum-GGT group. This study suggests a possible link between alcohol consumption and IgE sensitization, moreover, the risk of IgE sensitization was significantly higher in male high-risk drinkers. Therefore, clinicians should consider the risk of IgE sensitization possibly afflicting male high-risk drinkers.

## Introduction

Allergic diseases are immunoglobulin E (IgE)-mediated inflammatory diseases including asthma, allergic rhinitis, atopic dermatitis, food allergy and other allergic diseases. According to industrialization and westernization, the prevalence of allergic diseases has rapidly increased during recent decades in worldwide areas. Allergic diseases impose a considerable socioeconomic burden on sufferers and reduce their quality of life^1,2^.

IgE is the antibody class which binds to and activates effector cells, mast cells and basophils via the high affinity IgE receptor, FϲεRI. IgE is vital for allergic diseases, being an initiator of allergic reactions. Serum total and allergen-specific IgE levels are increased in allergic diseases^3,4^. Many factors other than allergic diseases affect the synthesis of IgE, such as parasite infections, smoking, liver diseases, primary immune deficiencies, age, and sex^5,6^.

Alcohol consumption is one of the top five risk factors for the global burden of disease, particularly heavy drinking^7^. Alcohol consumption is one of the most commonly experienced social pressures among Korean adults^8^. Worldwide alcohol consumption in 2015 was projected to be 6.3 liters of pure alcohol per person aged 15 years or older. Korea had the relatively high overall alcohol consumption (10.9 liters of pure alcohol per person)^9^.

Alcohol is a potent immunomodulatory agent, and its consumption has been linked to an increased risk of allergic diseases and elevated serum IgE level^10,11^, although other studies reported no association between alcohol consumption and allergic diseases^12^ or IgE sensitization^13^. In a recent study, observational analysis showed the associations of ever-drinking and allergic diseases. However, the results of Mendelian randomization suggest these associations were non-causal^14^. This discrepancy might be due to differences in alcohol exposure, laboratory methods, sample size, sociodemographic characteristics, and environmental factors among studies.

A considerable body of evidence supports sex differences in the prevalence of alcohol-use disorders and the physical effects of alcohol^15^, as well as in alcohol consumption, metabolism, and effects on the brain and certain receptor systems^16^. Cross-cultural comparisons generally report that the biological mechanisms underpinning the sex-specific differences in the effects of alcohol and related problems are independent of cross-country and cross-generational influences^17^. Also, sex differences in IgE sensitization to aeroallergens have been reported in the general population^18^. However, no study has examined sex differences in the association of alcohol drinking with the serum IgE level.

High-risk drinkers can be identified using the Alcohol Use Disorders Identification Test (AUDIT) questionnaire, which comprehensively assesses alcohol abuse^19,20^. The AUDIT questionnaire is a reliable test to measure high-risk across sex, age, and cultures^21,22^, underscoring its appropriateness for our study. However, as AUDIT is a subjective instrument for screening high-risk drinkers, its accuracy may be limited by poor respondent memory due to high alcohol consumption. Serum gamma-glutamyl transferase (GGT) is a liver enzyme that catalyzes the transfer of gamma-glutamyl groups to other amino acids and is a useful surrogate marker for excessive alcohol consumption^23^. We used the serum level of GGT as a marker of alcohol exposure and divided the subjects into three groups according to the sex-specific GGT tertile.

To our knowledge, no prior study has investigated the association of alcohol consumption pattern and the serum GGT level with IgE sensitization in a large population, or in one stratified by sex. The purposes of this population-based study of Korean adults were to 1) investigate the association between alcohol consumption and sensitization to specific allergens and 2) assess whether these associations differ by sex.

## Results

### Sociodemographic and clinical characteristics

The sociodemographic characteristics of the study population (unweighted number of participants, 918 males and 805 females) are listed in Table 1. The male and female subjects were divided into low-, intermediate-, and high-risk drinkers based on their AUDIT scores. Males constituted a higher proportion of the intermediate-risk (33.1% vs. 12.9%) and high-risk (23.3% vs. 3.7%) drinkers than females (p for trend < 0.0001) (Supplementary Fig. S1). The mean age was highest in male high-risk drinkers and female low-risk drinkers. For male groups, the proportions of high-risk drinkers were lowest in young adults and the elderly, and highest in those aged 41 to 50 years, generally showing a U-shaped distribution (Supplementary Fig. S2). Among females, the proportions of intermediate- and high-risk drinkers tended to decrease with age. High-risk drinkers of both sexes were more likely to be employed. In addition, high-risk drinkers of both sexes tended to be current smokers. Female high-risk drinkers were most likely to be obese and have no spouse, but no such findings were evident in males. In subgroup analyses, no significant differences were found in urban residence, educational level, income, sleep duration, or serum vitamin D level among the three risk groups.

**Table 1:** Sociodemographic and clinical characteristics by sex and AUDIT score.

Audit	Male				Female			
	Low	Inter-mediate	High	P value	Low	Inter-mediate	High	P value
**Numbers (%)**	400 (43.6)	304 (33.1)	214 (23.3)		671 (83.4)	104 (12.9)	30 (3.7)	
**Age (years)**	40.4 ± 0.9	37.3 ± 0.9	43.1 ± 0.9	<0.0001^*^	40.4 ± 0.5	33.5 ± 1.6	32.0 ± 1.8	<0.0001^*^
**Urban**	72.1	71.2	69.2	0.840	74.8	68.5	72.8	0.576
**Educational level (≤middle school)**	16.4	13.3	19.2	0.254	24.1	20.2	15.6	0.485
**Without spouse**	5.9	4.5	10.7	0.178	13.0	25.9	34.1	0.026^*^
**Income (lower quartile)**	38.9	37.6	44.1	0.486	43.6	37.0	45.0	0.603
**Without occupation**	26.2	21.8	12.4	0.005^*^	44.2	36.8	16.1	0.014^*^
**Current smoking**	40.8	57.5	62.0	<0.0001^*^	4.0	15.0	28.9	<0.0001^*^
**Regular exercise**	20.2	24.3	17.5	0.290	24.5	27.3	29.9	0.725
**Short sleep duration (≤6 hours)**	42.0	43.2	46.4	0.665	35.3	43.9	53.8	0.128
**BMI ≥ 25 kg/m** ^2^	34.8	37.6	40.5	0.531	23.4	14.8	53.6	0.001^*^
**Low vitamin D (≤20 ng/mL)**	38.6	36.9	44.0	0.418	21.4	33.6	25.1	0.065

Values are shown as percentages or means.

AUDIT: Alcohol Use Disorders Identification Test;

BMI: body mass index

*p < 0.05.

### Correlation between the AUDIT score and the serum GGT level

We examined the correlation between the AUDIT score and the serum GGT level using a general linear model. The AUDIT score had positive correlation with serum GGT level (coefficient (B): 2.626, 95% confidence interval (CI): 2.260–2.992). This correlation had statistical significance (R^2^ = 0.129, p < 0.0001).

### Allergy related factors according to alcohol consumption pattern

The prevalence of allergic disease did not differ according to alcohol consumption pattern in either sex (Table 2). Among males, the proportion of an increased total IgE level among high-risk drinkers (55.9%) was significantly higher than that in low- (38.6%) and intermediate-risk (44.7%) drinkers (p = 0.006). Similarly, among males, the frequency of atopy among high-risk drinkers (64.2%) was significantly higher than that in low-risk (49.9%) and intermediate-risk (54.2%) drinkers (Supplementary Fig. S3) (p = 0.028). Among females, there was no significant difference in the prevalence of an increased total IgE level or sensitization to specific allergens among the three alcohol consumption patterns.

**Table 2:** The percentage of subjects with allergic diseases and with an increased total or specific IgE by sex and AUDIT score.

Audit	Male				Female			
	Low	Inter-mediate	High	P value	Low	Inter-mediate	High	P value
**Numbers (%)**	400 (43.6)	304 (33.1)	214 (23.3)		671 (83.4)	104 (12.9)	30 (3.7)	
**Allergic diseases**								
**Asthma**	1.7	4.2	3.1	0.277	2.4	2.1	1.2	0.795
**Atopic dermatitis**	5.2	2.9	2.3	0.251	3.7	5.1	5.2	0.878
**Allergic rhinitis**	18.5	14.8	13.5	0.331	17.0	19.9	31.6	0.170
**Increased total/specific IgE**								
**Total IgE**	38.6	44.7	55.9	0.005^*^	21.0	26.4	38.9	0.123
**DF**	43.4	50.4	48.7	0.065	32.0	37.9	50.3	0.117
**Cockroach**	26.9	28.5	36.6	0.128	13.0	9.4	10.4	0.686
**Dog**	6.9	10.7	10.7	0.298	3.9	4.7	10.0	0.413
**Atopy**	49.9	54.2	64.2	0.027^*^	38.1	43.4	59.5	0.085

Values are shown as percentages or means.

AUDIT: Alcohol Use Disorders Identification Test; DF: *Dermatophagoides farinae*.

*p < 0.05.

### Associations between alcohol consumption pattern and total and specific IgE levels

**Figure 1:**
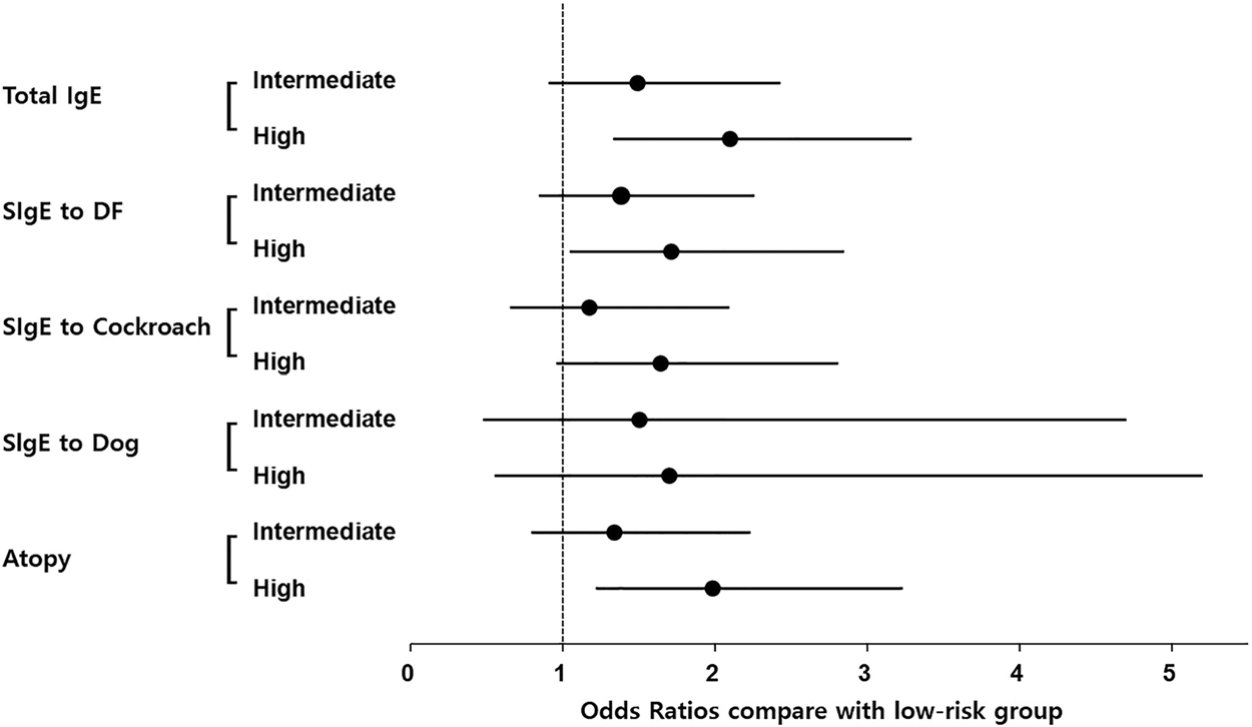
Associations of alcohol consumption with total and specific IgE levels in males. DF: *Dermatophagoides farina*; SIgE: specific IgE.

Table 3 shows the associations of alcohol consumption pattern with total and specific IgE levels by sex. In males, without adjustment, high-risk drinkers had a significantly increased OR for total and DF-, cockroach-specific IgE levels, and atopy compared with low-risk drinkers. After multivariable adjustment, the OR for total IgE, sensitization to DF, and atopy remained significant (Fig. 1).

**Table 3:** Associations of AUDIT scores with increased total IgE levels and IgE sensitization in males and females.

Audit	Male			Female		
	Low	Intermediate	High	Low	Intermediate	High
**Numbers (%)**	400 (43.6)	304 (33.1)	214 (23.3)	671 (83.4)	104 (12.9)	30 (3.7)
**Crude OR**						
Total IgE	1	1.45 (0.99–2.12)	2.01 (1.31–3.08)^*^	1	1.35 (0.75–2.42)	2.39 (1.03–5.58)^*^
DF	1	1.33 (0.90–1.96)	1.65 (1.05–2.60)^*^	1	1.29 (0.77–2.18)	2.14 (1.05–4.37)^*^
Cockroach	1	1.08 (0.72–1.61)	1.56 (1.01–2.42)^*^	1	0.69 (0.30–1.61)	0.78 (0.16–3.73)
Dog	1	1.61 (0.79–3.25)	1.60 (0.77–3.32)	1	1.20 (0.40–3.59)	2.74 (0.66–11.33)
Atopy	1	1.19 (0.81–1.74)	1.80 (1.16–2.81)^*^	1	1.24 (0.74–2.08)	2.39 (1.13–5.06)^*^
**Adjusted OR** ^†^						
Total IgE	1	1.49 (0.91–2.42)	2.09 (1.34–3.28)^*^	1	1.38 (0.68–2.80)	2.83 (0.98–8.15)
DF	1	1.38 (0.85–2.25)	1.71 (1.03–2.83)^*^	1	1.58 (0.83–3.00)	1.70 (0.63–4.63)
Cockroach	1	1.17 (0.66–2.08)	1.64 (0.96–2.80)	1	0.37 (0.09–1.48)	0.65 (0.12–3.54)
Dog	1	1.50 (0.48–4.69)	1.70 (0.56–5.19)	1	3.59 (0.91–14.16)	11.74 (2.04–67.24)^*^
Atopy	1	1.33 (0.80–2.22)	1.98 (1.22–3.22)^*^	1	1.42 (0.74–2.69)	1.84 (0.65–5.25)

^†^Multivariable adjustment for age, residential area, educational level, marital status, household income, occupation, smoking status, exercise status, sleep duration, obesity, vitamin D levels and allergic diseases.

AUDIT: Alcohol Use Disorders Identification Test; DF: *Dermatophagoides farinae*; OR: odds ratio.

*p < 0.05.

**Figure 2:**
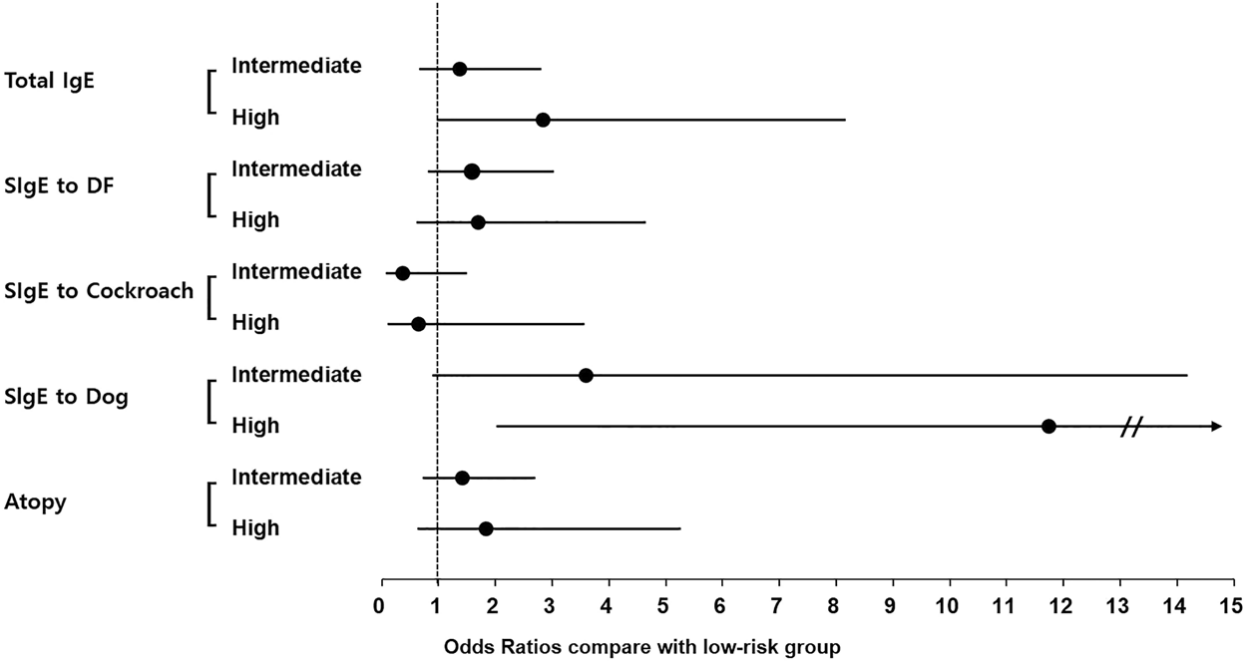
Associations of alcohol consumption with total and specific IgE levels in females. DF: *Dermatophagoides farina*; SIgE: specific IgE.

Without adjustment, female high-risk drinkers had a significantly increased OR for total and DF- and atopy-specific IgE levels compared with the low-risk drinkers. However, after multivariable adjustment, only the OR for dog-specific IgE level remained significant for female high-risk drinkers (Fig. 2).

### Associations between serum GGT levels and total and specific IgE levels

**Figure 3:**
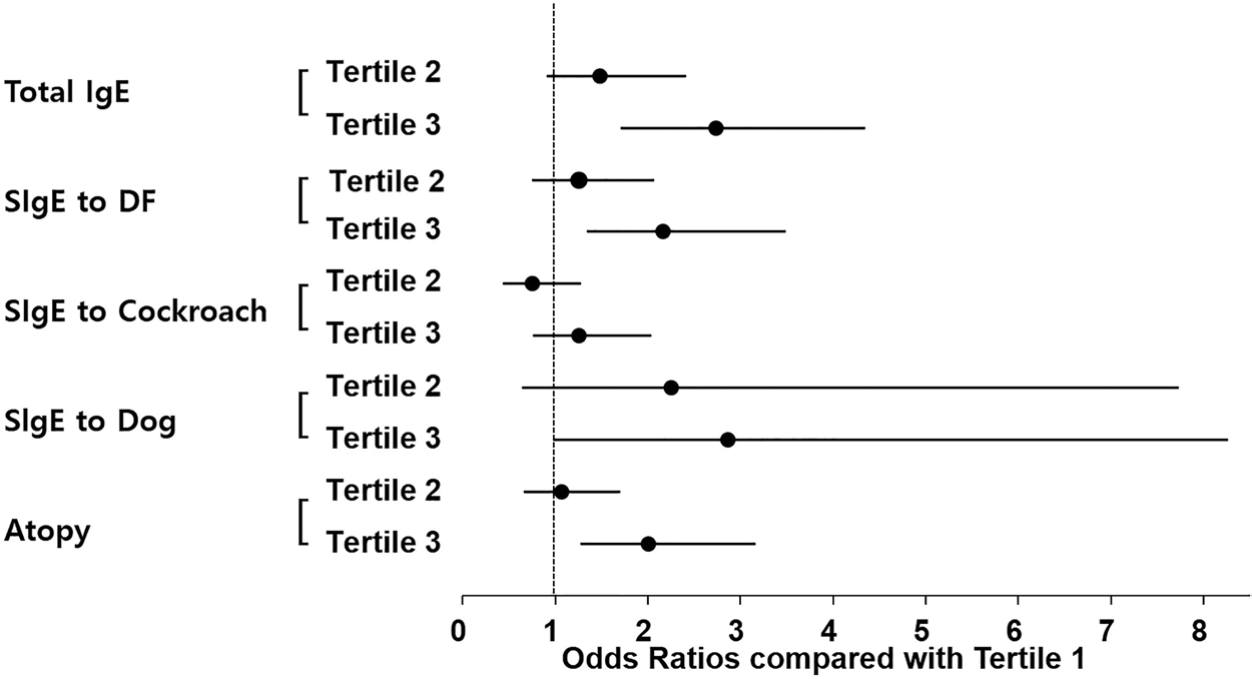
Associations of serum gamma-glutamyl transferase (GGT) level with total and specific IgE levels in males. DF: *Dermatophagoides farina*; GGT: gamma-glutamyl transferase; SIgE: specific IgE. Tertile: Sex-specific tertiles of serum GGT were used for analyses. In males, the tertile cut-off points were <25 IU/L (T1, tertile 1), 25 to 43 IU/L (T2, tertile 2), and >43 IU/L (T3, tertile 3).

Table 4 presents the associations of serum GGT tertiles with total and specific IgE levels by sex. Without adjustment, males in serum GGT tertile 3 showed a significantly increased OR for total and cockroach-specific IgE levels compared with those in tertile 1. After multivariable adjustment, the total and the DF-specific IgE and atopy were significantly associated with serum GGT tertile 3 but not with GGT tertile 1. The three IgE factors associated with a high serum GGT level were also associated with high-risk drinking (Fig. 3).

**Table 4:** Associations of serum GGT level with increased total IgE levels and IgE sensitization by sex.

GGT	Male			Female		
	T1^†^	T2^†^	T3^†^	T1^†^	T2^†^	T3^†^
**Numbers (%)**	311 (33.9)	303 (33.0)	304 (33.1)	300 (37.3)	250 (31.0)	255 (31.7)
**Crude OR**						
Total IgE	1	1.36 (0.93–1.98)	2.13 (1.44–3.16)^*^	1	1.20 (0.70–2.05)	1.73 (1.05–2.83)^*^
DF	1	1.01 (0.67–1.52)	1.33 (0.92–1.92)	1	1.21 (0.78–1.86)	0.97 (0.63–1.49)
Cockroach	1	1.11 (0.68–1.83)	1.75 (1.17–2.62)^*^	1	2.41 (1.27–4.53)^*^	1.74 (0.89–3.83)
Dog	1	1.32 (0.62–2.81)	1.39 (0.71–2.72)	1	0.94 (0.32–2.72)	1.07 (0.39–2.93)
Atopy	1	1.04 (0.68–1.57)	1.44 (0.99–2.11)	1	1.08 (0.71–1.64)	0.91 (0.58–1.43)
**Adjusted OR** ^‡^						
Total IgE	1	1.49 (0.91–2.40)	2.73 (1.72–4.33)^*^	1	1.25 (0.62–2.51)	1.24 (0.62–2.48)
DF	1	1.25 (0.76–2.06)	2.17 (1.35–3.47)^*^	1	1.07 (0.58–1.99)	0.92 (0.49–1.71)
Cockroach	1	0.75 (0.45–1.26)	1.25 (0.77–2.03)	1	2.02 (0.88–4.63)	1.62 (0.73–3.61)
Dog	1	2.25 (0.66–7.72)	2.86 (0.99–8.25)	1	2.31 (0.51–10.50)	3.67 (0.92–14.64)
Atopy	1	1.07 (0.67–1.69)	2.01 (1.28–3.15)^*^	1	0.87 (0.49–1.55)	0.95 (0.52–1.74)

^†^Sex-specific GGT tertiles based on their serum levels. In males, cut-off points were <25 IU/L (T (tertile) 1), 25 to 43 IU/L (T2), and >43 IU/L (T3). In females, the cut-off points were <14 IU/L (T1), 14 to 18 IU/L (T2), and >18 IU/L (T3).

^‡^Multivariable adjustment for age, residential area, educational level, marital status, household income, occupation, smoking status, exercise status, sleep duration, obesity, vitamin D levels and allergic diseases.

DF: *Dermatophagoides farinae*; GGT: gamma-glutamyl transferase; OR: odds ratio.

*p < 0.05.

The English in this document has been checked by at least two professional editors, both native speakers of English. For a certificate, please see: http://www.textcheck.com/certificate/J8ex65.

**Figure 4:**
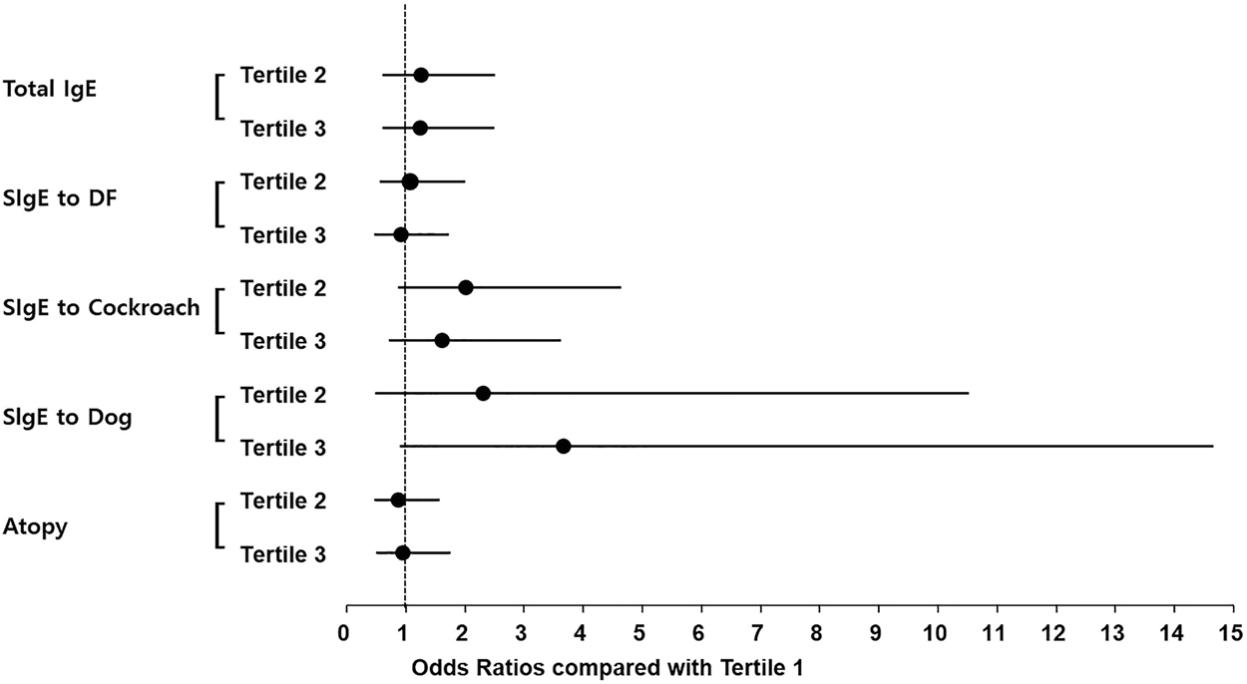
Associations of serum GGT level with total and specific IgE levels in females. DF: *Dermatophagoides farina*; GGT: gamma-glutamyl transferase; SIgE: specific IgE. Tertile: Sex-specific tertiles of serum GGT were used for analyses. In females, the cut-off points were <14 IU/L (T1, tertile 1), 14 to 18 IU/L (T2, tertile 2), and >18 IU/L (T3, tertile 3).

Without adjustment, among females, GGT tertile 3 was significantly associated with the total IgE level, and GGT tertile 2 with the cockroach-specific IgE level, compared with GGT tertile 1. However, after multivariable adjustment, no IgE level was significantly associated with GGT tertile 2 or 3 in females (Fig. 4).

## Discussion

This study in the general population examined the associations of alcohol consumption and serum GGT level with IgE sensitization by sex. Male high-risk drinkers had increased total and DF-specific IgE levels and a higher prevalence of atopy, while female high-risk drinkers had a high dog-specific IgE level, after adjusting for all confounders. Furthermore, in males, the three IgE factors related to high-risk drinking were associated with a high serum GGT level. In females, no IgE was associated with a moderate or high serum GGT level.

Previous studies of the association between alcohol consumption and allergic diseases and/or IgE sensitization have yielded inconsistent results. Moderately high alcohol consumption, as well as alcoholism, may be associated with high IgE levels and allergic disease^12,24,25^. Coutinho *et al*. reported that the serum total IgE level decreased significantly after cessation of alcohol consumption in Portuguese adults^26^. However, the association between alcohol consumption and IgE sensitization is less clear^13,27^. These inconsistent results might be caused by differences in alcohol exposure, laboratory methods, sample size, allergen sensitization profile, lifestyle, sex, and ethnicity among prior studies. Our findings suggest the importance of sex differences in the association between alcohol consumption and IgE sensitization.

The association of alcohol use with the GGT level has been demonstrated at the population level. A population-based study in Germany examined the associations of liver enzyme levels (as indicators of alcohol exposure) with the serum total IgE level. Alcohol consumption and the levels of liver enzymes, including that of GGT, were positively associated with the serum total IgE level in non-atopic and atopic subjects, consistent with our results^28^. However, the association of liver enzymes with the levels of allergen-specific IgE was not evaluated. Our findings of a significant association between both alcohol consumption pattern and the serum GGT level and the same three IgE factors (increased total and DF-specific IgE levels and the presence of atopy) strongly suggest that high-risk drinking is significantly associated with IgE sensitization in males.

Increased total IgE levels in heavy drinkers should be interpreted with caution because of the possible presence of specific IgE to cross-reactive carbohydrate determinants (CCDs)^29^. CCDs are N-glycans of plant and invertebrate glycoproteins that induce widespread IgE reactivity and interfere with *in vitro* tests for allergy^30,31^. CCD-specific IgE cross-reacts with IgEs to other aeroallergens, such as pollens and *Hymenoptera* venom^32^; its level is reportedly elevated in heavy drinkers^33^. Therefore, an increased total or specific IgE level alone is not indicative of allergy in drinkers. However, a recent study involving a large population by Vidal *et al*., predominantly assessing mite allergy patients, showed that CCD sensitization had no significant association with age, residential area, alcohol consumption, or smoking, and that CCD sensitization minimally interfered with IgE test results^34^. Also, CCD cross-reacts with pollens, latex allergens, *Hymenoptera* venoms and some food allergens, but to a lesser degree to DF, cockroach, and dog allergens^27,29^. Therefore, IgE associated with CCD likely had little influence on our results.

The mechanisms by which excessive alcohol consumption increases IgE levels are unclear^34^. Alcohol consumption impairs T-helper-type 1 (Th1) immune responses and increases T-helper-type 2 (Th2) responses in human^35,36^ and mouse^37,38^. Alcohol intake induces increased intestinal permeability and facilitates access of foreign materials such as microorganisms to the immune system. Alcohol also reduces host defenses and oral tolerance, and induces sensitization^39,40^. Heinz *et al*. proposed a unifying hypothesis of the mechanisms underlying the effect of alcohol on dendritic cells and alterations of adaptive immune responses. They demonstrated that alcohol consumption decreased the capacity of dendritic cells to produce Th1 cytokines, but enhanced Th2 cytokine production by CD4^+^ T cells^38^. However, Oldenburg *et al*. demonstrated that alcohol exposure reduced not only airway hyper-responsiveness, but also the serum IgE level and influx of airway eosinophils in a mouse model of allergic asthma^41^. Therefore, alcohol consumption modulates immunity in a general sense, rather than simply inducing a Th2 response.

Few studies have focused on sex differences in the association of alcohol consumption with IgE sensitization to aeroallergens. Sex differences in total IgE or allergen sensitization have in turn been attributed to sex differences in alcohol consumption^36,42^. Linneberg *et al*. reported that males had a higher alcohol intake and total IgE level than females in a population-based study of Danish adults^36^. Sex differences in immune responses according to alcohol consumption have been investigated using mouse models. Grossman *et al*. reported that alcohol significantly increased the population of CD4^+^ T-helper cells in male, but not female, mice^43^. On the other hand, Alonso *et al*. reported that alcohol administration increased the serum IgE and IL-13, but not IgA or IgM, levels in mice of both sexes^37^.

Female sex hormones stimulate allergic reactions, while little is known about the effects of androgens on the immune system^18,44^. However, the mechanisms underlying sex differences in allergic reactions are poorly understood^44^. Two *in vitro* studies reported that estradiol induces human mast cells to release allergic mediators and enhances IgE-mediated mast-cell activation and granulation^45,46^. In a rat model of allergic lung inflammation, the estradiol receptor antagonist tamoxifen ameliorated allergic lung inflammation^47^. Importantly, estrogen and estrogenic compounds increase IgE production by mouse splenocytes *in vitro*, and therefore may affect allergic sensitization^48^. Chronic alcohol use decreases testosterone level and increases estrogen level in males^49^ and increases testosterone and estradiol levels in females^50^. Our finding of a positive association between high-risk drinking and increased IgE levels in males may be attributed to increased estrogen levels due to high-risk drinking. Increased estrogen levels in high-risk males may lead to increased IgE production and allergic inflammation. However, because the KNHANES did not gather the data on sex hormones, we could not analyze the association between the serum levels of sex hormones and the serum IgE levels by alcohol consumption and sex. Further studies of sex differences in alcohol consumption and allergic factors, which take into consideration the serum levels of sex hormones, are warranted.

This study had several strengths. First, the KNHANES is a nationwide, population based survey, and the participants are representative of the general Korean population. Second, the serum levels of specific IgE were examined in a relatively large number of subjects. Third, we considered demographic characteristics, socioeconomic status, and comorbidities, and minimized bias by adjusting for confounding effects. Fourth, both subjective (AUDIT questionnaire) and objective (serum GGT level) measures of alcohol consumption were used. Fifth, this is the first study of sex differences in the association between alcohol consumption and IgE sensitization.

This study also had several limitations. First, a cross-sectional study cannot analyze the causal relationships between alcohol consumption and sensitization to aeroallergens. Second, parasite infection, which influences serum IgE levels, was not considered as a confounding factor, because the KNHANES does not include data on parasite infection. Third, despite the relatively large sample size, some of the female AUDIT score subgroups had few subjects (*e.g*. the number of female participants in the high AUDIT group = 30), resulting in wide confidence intervals. To overcome this problem, we used the sex-specific serum GGT tertiles. Fourth, based on the significant effect of both genetic variants on alcohol metabolism in populations of East Asian^51^ and European^52^ descent, our findings cannot be generalized to other populations. However, the ethnic homogeneity of the study population may reduce the influence of possible confounding variables. Further studies using data from the general populations of the United States and European nations are needed.

In conclusion, to our knowledge, this is the first study of the associations between alcohol consumption pattern and the serum GGT level with sensitization to specific allergens in the general population stratified by sex. Our findings suggest that high-risk alcohol consumption has a greater impact on IgE sensitization in males than in females. Therefore, the risk of IgE sensitization should be considered in patients who indulge in high-risk drinking, particularly in males.

## Methods

### Study population

The Korean National Health and Nutrition Examination Survey (KNHANES) is a nationwide, population-based, cross-sectional health examination survey, conducted by the Division of Chronic Disease Surveillance under the auspices of the Korea Centers for Disease Control and Prevention (KCDC). The KNHANES is conducted to evaluate the health and nutritional status of non-institutionalized Korean citizens. The survey consists of three components: a health interview, a nutritional survey, and a health examination survey. The health interview and health examination are performed by trained medical staff and interviewers in mobile examination centers. The nutritional survey is conducted in the homes of subjects 1 week after the health interview^53,54^. Peripheral blood samples were collected after fasting for more than eight hours, and were immediately processed and transported to a central laboratory (Neodin Medical Institute, Seoul, South Korea). All blood samples were analyzed within 24 hours of collection^55^.

We used data collected during the 2010 KNHANES. Of the 6,740 adults (≥19 years of age) who participated in the survey, 1,723 completed the clinical examination, the health questionnaire, and measurement of total/specific IgE (Supplementary Fig. S4). All subjects provided written informed consent for use of their data prior to survey commencement. The study was approved by the Institutional Review Board of the Korea Centers for Disease Control and Prevention (KCDC) (numbers 2010–02CON-21-C)^53^ and was performed in accordance with relevant guidelines/regulations.

### Alcohol consumption

The AUDIT questionnaire comprises three conceptual domains: hazardous alcohol use (frequency of drinking, typical quantity, and frequency of heavy drinking), dependence symptoms (impaired control over drinking, increased salience of drinking, and morning drinking), and harmful alcohol use (guilt after drinking, blackouts, alcohol-related injuries, and other concerns about drinking). The AUDIT scores were categorized into three groups according to the World Health Organization (WHO) guidelines: low risk, 0–7 points; intermediate risk, 8–15 points; and high risk, ≥16 points^22^.

The serum GGT level is a highly sensitive metric of alcohol consumption^56^ and the most widely used biomarker of alcohol abuse^23,57^. Serum GGT levels were assayed using a Hitachi Automatic Analyzer 7600 (Hitachi 7600, Tokyo, Japan). Male and female subjects were divided into tertiles based on their serum GGT level. The GGT levels of males were higher than those of females. Thus, sex-specific serum GGT tertiles were used in the analyses. In males, the cut-off points were <25 IU/L (tertile 1), 25 to 43 IU/L (tertile 2), and >43 IU/L (tertile 3). In females, the cut-off points were <14 IU/L (tertile 1), 14 to 18 IU/L (tertile 2), and >18 IU/L (tertile 3).

### Measurement of total and specific IgE levels

Serum samples were tested for total IgE and allergen-specific IgE using a 1470 WIZARD-ɣ-Counter analyzer (PerkinElmer, Turku, Finland) with ImmunoCAP100 (Phadia, Uppsala, Sweden). The *Dermatophagoides farinae* ([DF] house dust mite)−, *Blattella germanica* (German cockroach)- and dog-specific IgE levels were measured. Only these three allergen-specific IgEs were included in the KNHANES data. An increased total IgE level was defined as a serum level of ≥150 kU/L^58^. The cut-off value for serum aeroallergen-specific IgE level was 0.35 kU/L^59^.

### Definitions of allergic diseases and atopy

All subjects were asked about their history of allergic diseases. The lifetime prevalence of allergic diseases, including asthma, allergic rhinitis, and atopic dermatitis, was assessed by a physician. Atopy was defined as a level of IgE against at least one of the three aeroallergens of 0.35 kU/L or greater^60^.

### Sociodemographic characteristics and lifestyle factors

During the health interviews, data were collected using self-reported questionnaires. The health interview evaluated educational level, occupation, household income, residential area, marital status, smoking, exercise status, and sleep duration. Smoking history was categorized as current smoker or nonsmoker. Regular exercise was defined as moderate physical activity performed for at least 30 minutes at a time at least five times a week. Height, weight, and waist circumference were measured by trained medical professionals^53^. The body mass index (BMI) was calculated as body weight (kg) divided by height in meters squared (m^2^). Obesity was defined as a BMI ≥ 25 kg/m^2^ ^61^.

### Statistical analysis

Statistical analyses were performed using the complex sample analysis procedure of PASW software (ver. 18.0; SPSS Inc., Chicago, IL, USA) to reflect the complex sampling design and sampling weights of the KNHANES, and to provide nationally representative prevalence estimates. Results are presented as percentages ± standard error for categorical variables and as estimated means ± standard error for continuous variables. To compare categorical and continuous variables, the chi-squared test and a general linear model, respectively, were used. The multivariable analysis employed logistic regression to explore the associations of alcohol consumption with IgE sensitization. We calculated the odds ratios (ORs) for IgE sensitization and atopy according to sex. Multivariable adjustment was for age, residential area, educational level, marital status, household income, occupation, smoking status, exercise status, sleep duration, obesity, vitamin D level and allergic diseases, because these variables are reportedly associated with the serum IgE levels, alcohol consumption, or both^53^.

### Previous presentation

This study was presented at the European Academy of Allergy and Clinical Immunology (EAACI) Congress 2018; 29 May 2018.

## Supplementary information

Supplementary information

## Data Availability

The authors used 2010 KNHANES data. All files are available from the KNHANES webpage (https://knhanes.cdc.go.kr/knhanes/eng/index.do;jsessionid=U1mFHXN2dzfKNNVOvQKB0nmYaQqn05mBwm108CngA0EMjVKaMeFyOGtRg1Eelh7d.KCDCWAS01_servlet_PUB1). All other relevant data are within the paper and its Supporting Information files.
