# Supplementary material for: Sex difference in IgE sensitization associated with alcohol consumption in the general population
Source: Sci Rep. 2019 Aug 20;9:12131. doi: 10.1038/s41598-019-48305-y (PMC6702201; doi:10.1038/s41598-019-48305-y)

Supplementary information

Sex difference in IgE sensitization associated with alcohol consumption in the general population

Daeyoung Roh[1], Dong-Hee Lee[2], Sang-Kyu Lee[1], Soo Whan Kim[2], Sung Won Kim[2], Jin Hee Cho[2], Byung-Guk Kim[2], and Ji-Hyeon Shin[2,*]

[1] Mind-neuromodulation Laboratory and Department of Psychiatry, Chuncheon Sacred Heart Hospital, Hallym University College of Medicine, Chuncheon-si, Gangwon-do, Republic of Korea

[2] Department of Otolaryngology-Head and Neck Surgery, College of Medicine, The Catholic University of Korea, Seoul, Republic of Korea

Supplementary Figure S1. Distribution of by AUDIT groups (low-, intermediate-, and high-risk groups) by sex

AUDIT: Alcohol Use Disorders Identification Test

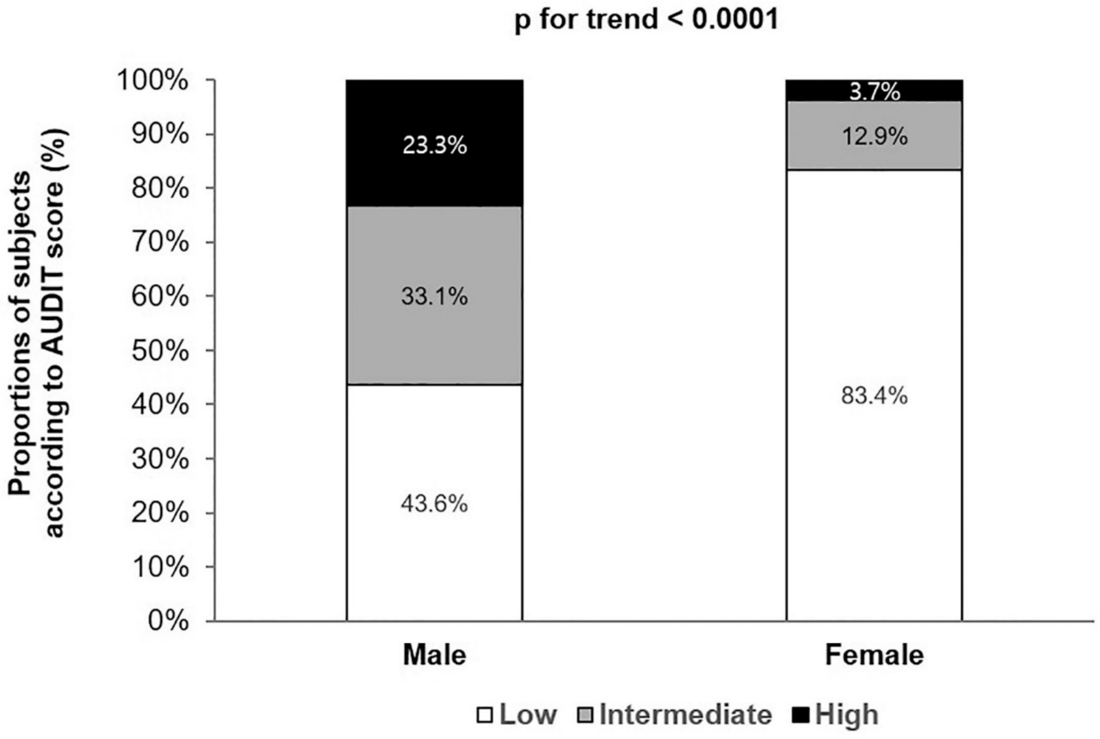

Supplementary Figure S2. Distribution of by AUDIT groups (low-, intermediate-, and high-risk groups) by age in males and females

AUDIT: Alcohol Use Disorders Identification Test

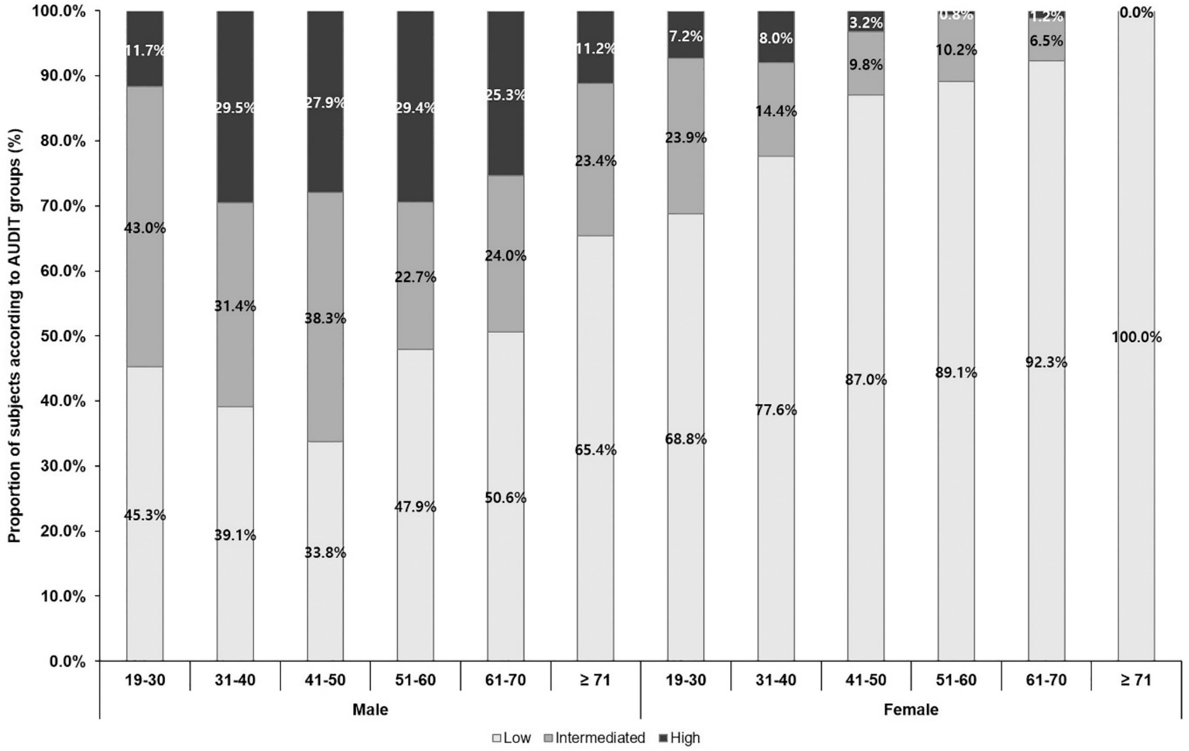

Supplementary Figure S3. Frequencies of increased total, house-dust mite-, cockroach-, and dog-specific immunoglobulin (IgE) levels, as well as atopy, by AUDIT score in males and females

AUDIT: Alcohol Use Disorders Identification Test; DF: *Dermatophagoides farina*

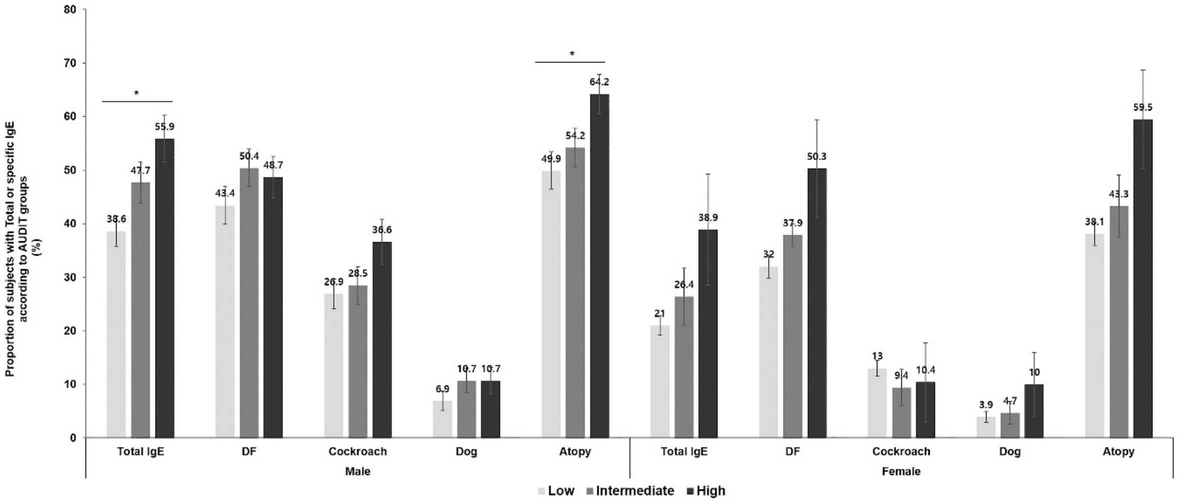

Supplementary Figure S4. Flow chart showing the selection of study participants.

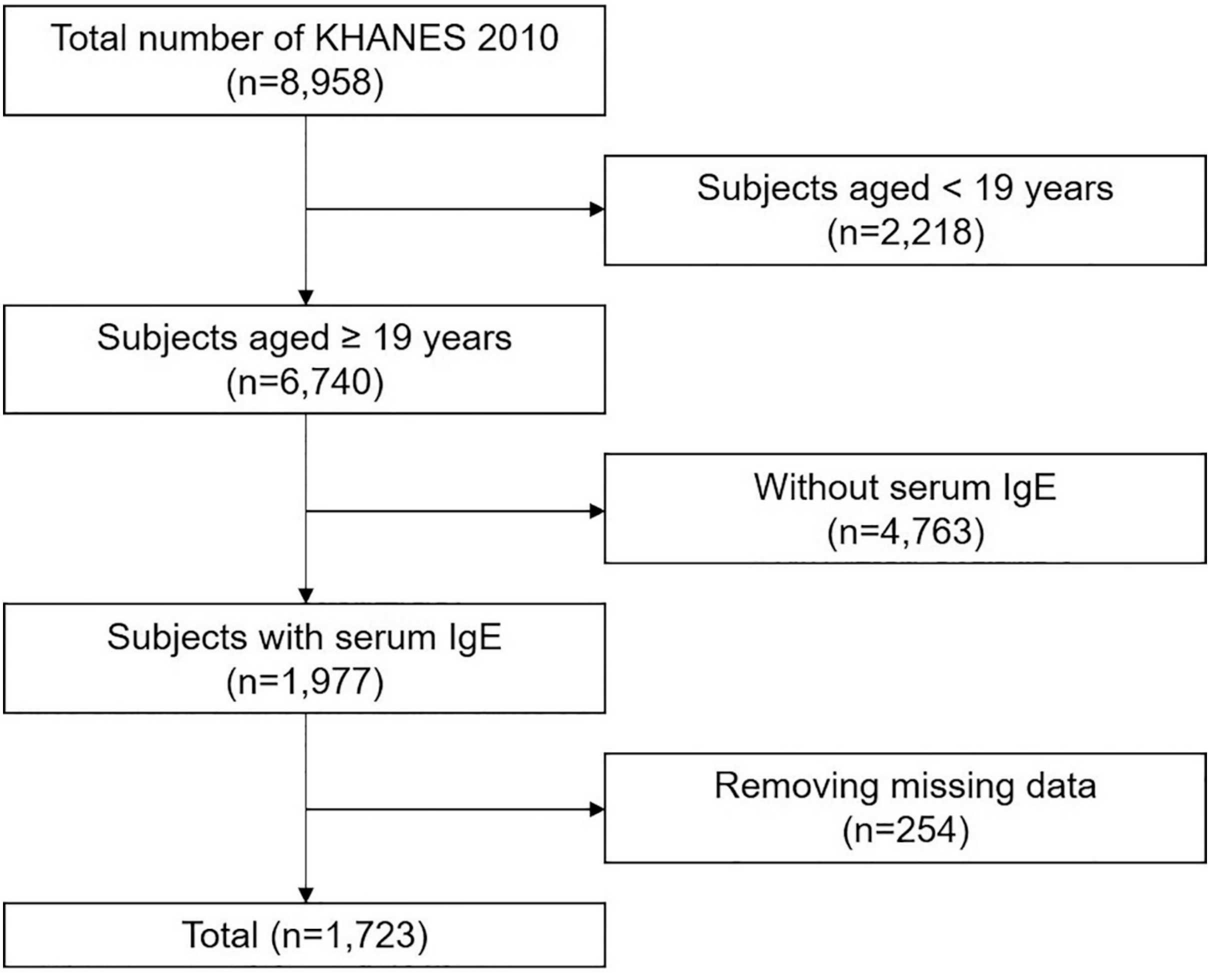

Supplement: Supplementary file 1 — Supplementary information [file 41598_2019_48305_MOESM1_ESM.pdf]
